# Calcineurin inhibitors in the treatment of primary focal segmental glomerulosclerosis: A protocol of systematic review and meta-analysis of randomized controlled trials

**DOI:** 10.1097/MD.0000000000024533

**Published:** 2021-01-29

**Authors:** Guozhong Xue, Xinbin Wang, Shuwen Li, Enlai Dai

**Affiliations:** Gansu University of Chinese Medicine, Lanzhou, China.

**Keywords:** calcineurin inhibitors, meta-analyses, primary focal segmental glomerulosclerosis, randomized controlled trials, systematic review

## Abstract

**Background:**

Evidence suggesting a role for including calcineurin inhibitors(CNIs) in early therapy remains limited for low quality and mainly based on small observation cohort study. We will conduct a systematic reviews to explore the effect and adverse effect of calcineurin inhibitors compared with other interventions in the treatment of primary focal segmental glomerulosclerosis (FSGS).

**Methods:**

A comprehensive literature search of MEDLINE (through PubMed), EMBASE, The Cochrane Library, Cochrane Central Register of Controlled Trials (CENTRAL) will be conducted. Two investigators will independently select studies, extract data and assess the quality of the included study. Extracted information will include study characteristics, the contents of included randomized controlled trials, outcomes, the quality of randomized controlled trials and etc. A risk of bias tool will be used to assess the methodological quality. Any disagreement will be resolved by the third investigator. There is no requirement of ethical approval and informed consent.

**Results:**

This study will provide high-quality evidence for treatment of FSGS in terms of effectiveness and safety.

**Conclusion:**

This systematic review aims to provide evidence for treatment of FSGS in different CNIs.

**Registration:**

The systematic review and meta-analysis is registered in the OSF REGISTERS (10.17605/OSF.IO/3B7DE) international prospective register of systematic review.

## 1 Introduction

Primary focal segmental glomerulosclerosis (FSGS) most commonly presented with nephrotic syndrome and the associated features of peripheral edema, hypoalbuminemia, and, usually, high-grade (>3.5 g/d) proteinuria.^[1,2]^ Studies evaluating the clinical characteristics of patients with primary FSGS have found that 70 to nearly 100 percent present with nephrotic syndrome.^[3,4]^

Calcineurin inhibitors (CNIs; cyclosporine or tacrolimus) are used with or without low-dose prednisone as initial therapy in patients at increased risk for glucocorticoid-associated toxicity (e.g., obese patients, diabetic patients, patients with severe osteoporosis, patients > 70 years of age).^[5,6]^ Evidence suggesting a role for including CNIs in early therapy remains limited for low quality and mainly based on small observation cohort study.^[5,7]^ A Cochrane systematic review performed in 2008 concluded that the number of primary FSGS patients with complete or partial remission in the cyclosporine and low prednisone group than patients treated with symptomatic treatment or prednisolone alone.^[8]^ A systematic review and meta-analysis performed in 2017 in order to review the efficacy of CNIs in the treatment of primary FSGS. However, the small number of included studies and treatment protocols’ heterogeneity and potential publication bias limited their conclusion.^[9]^ Thus, the effect of CNIs in the treatment of primary FSGS is still unclearly.

As we all known, high quality meta-analyses has been increasingly regarded as one of the key tools for achieving evidence.^[10–12]^ The purpose of this systematic review and meta-analysis is to summarize the existing randomized controlled trials (RCT) regarding the effect and adverse effect of CNIs compared with other interventions in the treatment of primary FSGS, both as first-line therapy and as an adjunctive agent in corticosteroid therapy resistant patients.

## 2 Method

This meta-analysis had applied for registration in the OSF REGISTERS (10.17605/OSF.IO/3B7DE). The study will be conducted according to the preferred reporting items for systematic reviews and meta-analysis protocols guidelines.^[13,14]^ No requirement of ethical approval and informed consent are needed because it is a retrospective study.

### 2.1 Search strategy

We will conduct a comprehensive systematic search to identify all relevant published studies from inception to November 10, 2020.^[15]^ There will be no restriction on the publication dates and languages for search. The following electronic databases will be searched: MEDLINE (through PubMed), EMBASE, The Cochrane Library, Cochrane Central Register of Controlled Trials (CENTRAL). The search strategy will combine MeSH and full text terms relating to the populations (i.e., glomerulosclerosis, focal segmental), interventions (i.e., calcineurin inhibitor, tacrolimus, cyclosporine). To identify other relevant study data, we will contact the authors of published studies for incomplete data.

### 2.2 Inclusion criteria

We will include studies which met the following criteria:

1) Types of participants
  We will include adult patients with primary FSGS without limitation on other aspects.
2) Types of interventions
  Calcineurin Inhibitors (i.e., tacrolimus, cyclosporine) was used as an intervention of disease.
3) Types of comparations
  The comparations were not limited.
4) Types of outcomes
  The primary outcome was mortality. The secondary outcomes were renal survival/time to end stage kidney disease, proteinuria remission rate (partial and complete), and renal function (estimated glomerular filtration rate).
5) Types of studies
  Randomized controlled trials.

### 2.3 Exclusion criteria

Studies will be excluded if one of the following conditions is met:

1) the research data was not complete;
2) the studies were cross section studies, case-control studies, case reports, case series and briefs, comments, letters, conference abstracts, editorials, protocols, nonhuman studies;
3) the study was not in English and Chinese.

### 2.4 Study selection

Two investigators will independently select the studies and any disagreement will be resolved by a third investigator through discussion. Titles and abstracts of the studies retrieved by the literature search will be screened base on inclusion/exclusion criteria and we will acquire the full text of potentially relevant studies for further assessment.

### 2.5 Data extraction

A standard form will be used to extract data from the included studies. Two investigators will independently extract the related data and any dispute will be discussed and resolved by a third investigator. Missing data will be requested from study authors. Extracted information will include:

1) study characteristics: first author, publication year, journal, country of origin and funding source;
2) the contents of included RCTs: sample size, mean age of participants, gender, specific condition), characteristics of interventions and comparators;
3) outcomes: mortality, renal function, proteinuria remission, and incidence of side effect;
4) assess the quality of RCTs: the item of risk of bias (ROB).

### 2.6 Quality evaluation

Two reviewers will use the Cochrane ROB tool^[16,17]^ to assess included studies. The risk of bias tool covers 6 domains: selection bias (methods of randomized sequence generation, and allocation concealment), performance and detection bias (the presence or absence of blinding), attrition bias (levels and reporting of loss to follow-up), reporting bias (reporting bias due to selective outcome reporting), and other bias (bias due to problems not covered elsewhere). We will apply the grades of recommendation, assessment, development and evaluation system to assess quality of the evidence.^[18,19]^

### 2.7 Statistical analysis and assessment of heterogeneity

Review Manager 5.3 software and Stata 15.0 will be used for general meta-analysis.

#### 2.7.1 Measures of treatment effect

We will use a random-effects model to pool the results of all studies, the relative risk and 95% confidence intervals will be used for summarizing individual trial outcomes and for estimates of pooled effect. If data were not reported in a form that would allow inclusion in the meta-analysis, we will report the results narratively.

#### 2.7.2 Assessment of heterogeneity

Cochran test and the I^2^ statistic will be used to evaluate the combination of heterogeneous studies. A I^2^ greater than 50% may be considered to indicate substantial heterogeneity. Subgroup analyses will be further conducted to investigate potential source of heterogeneity on treatment effect size, including clinical heterogeneity or methodological heterogeneity.

#### 2.7.3 Publication bias

Where the number of studies for an outcome is sufficient (n ≥ 10), a funnel plot will be used to examine for potential publication bias.

#### 2.7.4 Subgroup and sensitivity analysis

We plan to undertake subgroup analysis to examine effects of different comparisons, and populations when appropriate. When applicable, we will perform sensitivity analyses of results to look at the possible contribution of ROB such as randomization process, and concealment of random allocation.

## 3 Discussion

FSGS accounts for a significant proportion of nephrotic syndrome and is the most common cause of end-stage renal disease (ESRD) related to glomerular disease in adults.^[7,20,21]^ A study reported an annual incidence rates of FSGS as 0.2 to 1.8/100, 000 population per year by reviewing literature worldwide.^[22]^ Improving global out-comes (KDIGO) practice guidelines for the treatment of patients with FSGS suggests CNIs are considered for FSGS patients who developed steroid-dependent.^[23]^ The existing evidence suggests that CNIs in combination with glucocorticoids may increase the likelihood of complete or partial remission of proteinuria among patients with steroid-resistant primary FSGS.

This review will describe and analyze characteristics of the effect and adverse effect of CNIs as first-line therapy and second-line therapy compared with other interventions in the treatment of FSGS patients and provide a comprehensive summary of the existing RCTs on the immunosuppressive treatment for FSGS. And this review will apply the grades of recommendation, assessment, development and evaluation system to assess quality of the evidence.

Also, the lack of sample size of included studies and treatment protocols’ heterogeneity are some problems and limitations in this review. The final report of the systematic review and meta-analyses in the form of scientific paper will be published in peer-reviewed journals.

## Author contributions

**Conceptualization:** Guozhong Xue, Enlai Dai.

**Data curation:** Guozhong Xue, Xinbin Wang, Shuwen Li.

**Formal analysis:** Guozhong Xue, Xinbin Wang, Enlai Dai.

**Investigation:** Guozhong Xue, Xinbin Wang.

**Methodology:** Guozhong Xue, Xinbin Wang, Shuwen Li.

**Project administration:** Enlai Dai.

**Software:** Guozhong Xue, Xinbin Wang, Shuwen Li.

**Supervision:** Enlai Dai, Guozhong Xue.

**Writing – original draft:** Guozhong Xue.

**Writing – review & editing:** Xinbin Wang, Shuwen Li, Enlai Dai.

All authors approved the final version of the manuscript.

## Abbreviations

Abbreviations: CNIs = calcineurin inhibitors, FSGS = focal segmental glomerulosclerosis, RCTs = randomized controlled trials, ROB = risk of bias.
